# The ins and outs of eukaryotic viruses: Knowledge base and ontology of a viral infection

**DOI:** 10.1371/journal.pone.0171746

**Published:** 2017-02-16

**Authors:** Chantal Hulo, Patrick Masson, Edouard de Castro, Andrea H. Auchincloss, Rebecca Foulger, Sylvain Poux, Jane Lomax, Lydie Bougueleret, Ioannis Xenarios, Philippe Le Mercier

**Affiliations:** 1 SIB Swiss Institute of Bioinformatics, CMU, University of Geneva Medical School, Geneva, Switzerland; 2 European Molecular Biology Laboratory, European Bioinformatics Institute (EMBL-EBI), Wellcome Trust Genome Campus, Hinxton, United Kingdom; University of Padua, ITALY

## Abstract

Viruses are genetically diverse, infect a wide range of tissues and host cells and follow unique processes for replicating themselves. All these processes were investigated and indexed in ViralZone knowledge base. To facilitate standardizing data, a simple ontology of viral life-cycle terms was developed to provide a common vocabulary for annotating data sets. New terminology was developed to address unique viral replication cycle processes, and existing terminology was modified and adapted. The virus life-cycle is classically described by schematic pictures. Using this ontology, it can be represented by a combination of successive terms: “entry”, “latency”, “transcription”, “replication” and “exit”. Each of these parts is broken down into discrete steps. For example Zika virus “entry” is broken down in successive steps: “Attachment”, “Apoptotic mimicry”, “Viral endocytosis/ macropinocytosis”, “Fusion with host endosomal membrane”, “Viral factory”. To demonstrate the utility of a standard ontology for virus biology, this work was completed by annotating virus data in the ViralZone, UniProtKB and Gene Ontology databases.

## Introduction

What could be more alien than a virus? These parasitic entities evolve at the periphery of cellular organisms, and have developed unique methods to replicate and disseminate their genetic material. Many of these unique molecular processes may find their root in ancient biochemistry, down to the RNA world [1]. Indeed today cell’s genomes are all double stranded DNA (dsDNA), whereas viral genomes display all kinds of imaginable nucleic acid templates: single strand, double strand, DNA or RNA. Natural selection has privileged dsDNA cellular organisms, while keeping complete viral genomic diversity. Indeed this is advantageous to viruses, because their host cells have difficulty setting up antiviral defenses against that much diverse invading genetic material. This amazing viral diversity calls for various replication strategies: each kind of virus family has their own way of entering, replicating and exiting the host cell. But the number of unique viral processes is much lower than that because many virus families use similar means at different steps of the replication cycle.

In this work the SwissProt virus annotation team addressed the annotation and classification of all major means used by eukaryotic viruses to achieve their parasitic life-cycle. An extensive study of viral textbooks and the recent literature was performed to identify essential and conserved viral life-cycle steps. This study has focused on processes directly involved in entry, expression, replication and exit of the viral genetic material. Host-virus interactions implicated in immunity have been covered in previous publications [2,3]. Despite their large diversity, replication cycles can be described by a moderate number of different steps. The great diversity of replication cycles comes from the various combinations of these steps. For example there are 8 ways for viruses to cross the host membrane, 11 ways to replicate their nucleic acids, and more than 4 routes to exit the cell. A virus life-cycle can therefore be described by a succession of events. To further characterize this, we have created a controlled vocabulary comprising 82 terms that together cover all the major molecular events of a eukaryotic virus replication cycle.

The 82 terms describing the core viral replication cycle were used to annotate virus entries in ViralZone [4], UniProt [5] and Gene Ontology (GO) [6,7]. The annotation consists of associating viral sequences with experimental knowledge, and is expressed in the form of human-readable text, ontologies and controlled vocabularies which are searchable and even amenable to interpretation by machines. This requires human experts with deep knowledge of the underlying biology and a clear understanding of how to express and encode that knowledge in a consistent manner. Curators also perform an editorial function, acting to highlight (and where possible resolve) conflicting reports—one of the major added values of manual annotation. The processes identified have been developed in the form of controlled vocabulary and ontologies stored in the ViralZone, UniProtKB and GO resources.

ViralZone is a database that links virus sequence with protein knowledge using human-readable text and controlled vocabularies [4]. This web resource was created in 2009 and has been continually developed since that time by the viral curation team of the SwissProt group. The web site is designed to help people gain access to an abstraction of knowledge on every aspects of virology through two different kinds of entries: Virus fact sheets and virus molecular biology pages. The latter describe viral processes such as viral entry by endocytosis and viral genome replication in detail, with graphical illustrations that provide a global view of each process and a listing of all known viruses which conform to the particular schema. ViralZone pages also provide an access to sequence records, notably to the UniProt Knowledgebase (UniProtKB).

UniProtKB is a comprehensive resource for protein sequence and annotation data [5]. All known proteins are annotated in dedicated entries, either manually (Swiss-Prot) or automatically (TrEMBL). Annotation of protein function and features are assured by many means, including controlled vocabularies and ontologies. Ontologies consist of hierarchized controlled vocabulary in computer-friendly format. They provide a frame for global annotation, and facilitate analysis of biological data. In the era of metagenomics and large-scale studies, ontologies are an extremely potent tool to link knowledge with gene products and help identify common patterns. UniProtKB keywords constitute an ontology with a hierarchical structure designed to summarize the content of an entry and facilitate the search of proteins of interest. They are classified in 10 categories: Biological process, Cellular component, Coding sequence diversity, Developmental stage, Disease, Domain, Ligand, Molecular function, Post-translational modification and Technical term.

A more complex and widely used vocabulary is that of the Gene Ontology (GO) in which relations between terms have a number of explicit meanings which can be used to make further inferences–such as eukaryotic transcription factors may be located in the nucleus [6,7]. GO annotations are routinely used for the functional analysis (typically enrichment analysis) of many data types, such as differential expression data. GO provides almost 40,000 terms grouped in three categories: the molecular functions a gene product performs, the biological processes it is involved in and the cellular components it is located in. But until now, comprehensive eukaryotic virus biology has not been thoroughly described in this ontology. GO annotations are created manually, by expert curators, as well as by automatic propagation systems. The manual curation of GO terms is a central part of the workflow at UniProt, and UniProt is an active member of the GO consortium. Many UniProtKB keywords are also mapped to equivalent GO terms, and the occurrence of a keyword (KW) annotation allows the annotation of the equivalent GO term (http://www.ebi.ac.uk/GOA/Keyword2GO).

The virus replication cycle core terms have already been implemented in these three resources by over 12,000 manual and 2,000,000 automatic annotations. This work provides a basal knowledge of virus protein function that can be used as a reference for similar sequences, thereby facilitating analysis of large scale datasets with viral protein expression.

## Material and methods

This work describes the creation of a virus life-cycle vocabulary in ViralZone, UniProtKB and Gene Ontology. Inter-relations between vocabulary and ontologies, and the way virus sequences are curated using this system have been described in a previous publication [2].

### Creation of virus life-cycle vocabulary and ViralZone pages

The first step of this work was to identify all specific steps used by eukaryotic viruses during their life-cycle. To do so, an exhaustive review was performed in virology textbooks, published reviews, and existing ontologies by the UniProtKB/Swiss-Prot virus team. All the processes identified were structured into chronological steps involved in virus entry, transcription/replication/translation and exit. This led to the creation of 69 ViralZone pages describing most of the identified vocabulary (Table 1). The ViralZone pages were first annotated to describe the viral process, illustrated with a picture and the viruses involved were listed and linked to literature references. The controlled vocabulary resulting from this work is not hierarchical, but ordered chronologically for entry and exit. This work is the base used to build and refine ontologies in Gene Ontology and UniProtKB/Swiss-Prot.

**Table 1:** Virus life-cycle vocabulary.

Viral cycle vocabulary	UniProt KW	SwissProt annotation	TrEMBL annotation	ViralZone page	GO term	UniProt2GO annotation	GO annotation
**Viral life cycle**				VZ-873	GO:0019058		**2615722**
**VIRUS ENTRY**	**KW-1160**	**2'460**	**468'555**	**VZ-936**	**GO:0046718**	**471'015**	**1763463**
Viral attachment to host cell	KW-1161	1'349	390'329	VZ-956	GO:0019062	391'678	403'870
Viral penetration into host cytoplasm	KW-1162	1'389	379'148		GO:0046718	380'537	1'768'995
Fusion of virus membrane with host membrane	KW-1168	913	368'519		GO:0039663	369'432	618'339
*> Fusion of virus membrane with host cell membrane*	KW-1169	247	10'947	VZ-987	GO:0019064	11'194	128'441
*> Fusion of virus membrane with host endosomal membrane*	KW-1170	587	116'146	VZ-992	GO:0039654	116'733	118'210
Pore-mediated penetration of viral genome into host cell	KW-1172	44	0	VZ-979	GO:0044694	44	44
Apoptotic mimicry				VZ-5996			
Virus endocytosis by host	KW-1164	690	95305	VZ-977	GO:0075509	95'995	281'631
*> Caveolin-mediated endocytosis of virus by host*	KW-1166	56	0	VZ-976	GO:0075513	56	56
*> Clathrin- and caveolin-independent endocytosis of virus*	KW-1167	183	92362	VZ-978	GO:0019065	92'545	185'634
*> Lipid-mediated endocytosis of virus*				VZ-5496			
*> Clathrin-mediated endocytosis of virus*	KW-1165	448	92'810	VZ-957	GO:0075512	93'258	92'919
*> Macropinocytosis of virus*				VZ-800	GO:0075510		0
Viral penetration via lysis of endosomal membrane	KW-1174	12	0	VZ-984	GO:0039664	12	15
Viral penetration via permeabilization of endosomal membrane	KW-1173	134	7686	VZ-985	GO:0039665	7'820	7'820
Cell to cell transport	KW-0916	306	620	VZ-1018	GO:0046740	926	2'941
Cytoplasmic inwards viral transport	KW-1176	217	787	VZ-990	GO:0075733	1'004	56'518
*> Actin-dependent inwards viral transport*	KW-1178	4	0	VZ-991	GO:0075520	4	4
*> Microtubular inwards viral transport*	KW-1177	213	787	VZ-983	GO:0075521	1'000	2'477
Viral penetration into host nucleus	KW-1163	579	51518	VZ-989	GO:0075732	52'097	52'106
Viral genome integration	KW-1179	189	15835	VZ-980	GO:0075713	16'024	16'129
Viral factories				VZ-1951	GO:0039713		40
**VIRAL TRANSCRIPTION/REPLICATION**	** **	** **	** **	** **	** **	** **	** **
Viral DNA replication	KW-1194	79	0		GO:0039693	79	572
*> ssDNA rolling circle*				VZ-1941	GO:0039684		17
*> Rolling hairpin replication*				VZ-2656	GO:0039685		2
*> Bi -directional replication*				VZ-1939	GO:0039686		46
*> dsDNA rolling circle*				VZ-2676	GO:0039683		0
*> dsDNA strand displacement*				VZ-1940	GO:0039687		26
*> Circular reverse-transcription*				VZ-1938	GO:0039688		0
Viral RNA replication	KW-0693	893	100564		GO:0039694	101'457	101028
*> linear reverse-transcription*				VZ-1937	GO:0039692		36
*> dsRNA-templated transcription/replication*				VZ-1116	GO:0039690		59
*> dsRNA replication*				VZ-1936	GO:0039691		0
*> ssRNA-templated replication*				VZ-1096	GO:0039689		226
*> ssRNA rolling circle*				VZ-1944			
Viral transcription	KW-1195	213	59953		GO:0019083	60'166	99370
*> DNA templated transcription*				VZ-1942	GO:0039695		58
*> RNA templated transcription*				VZ-1936	GO:0039696		36631
*> Nested subgenomic transcription*				VZ-1876			
*> ssRNA(-) transcription*				VZ-1917	GO:0039697		36576
*> Hepatitis D transcription*				VZ-4116			
*> Cap snatching*	KW-1157	218	36337	VZ-839	GO:0075526	36'555	36555
*> Poly A stuttering*				VZ-1916	GO:0039698		0
*> Ambisens transcription*				VZ-1945			
*> RNA editing*	(KW-0691)			VZ-857 VZ-834	GO:0075527		0
*> Alternative splicing*	(KW-0025)			VZ-1943	GO:0000380		421
Early viral transcription	(KW-0244)				GO:0019085		4
middle viral transcription					GO:0019084		0
late viral transcription	(KW-0426)				GO:0019086		35
Viral Translation					GO:0019081		45
*> Viral initiation of translation*				VZ-867	GO:0075522		19
*> RNA suppression of termination*	(KW-1159)			VZ-859	GO:0039705		0
*> ribosomal skipping*	(KW-1197)			VZ-914	GO:0075524		0
*> Termination/reinitiation*	(KW-1158)			VZ-858	GO:0075525		11
*> Translational shunting*	(KW-1156)			VZ-608	GO:0039704		11
*> Leaky scanning*				VZ-1976			
**VIRUS EXIT FROM HOST CELL**	**KW-1188**	**208**	**16'084**	**VZ-1076**	** **	**16'292**	** **
Viral genome packaging	KW-0231	230	0		GO:0019072	230	1217
*> Cytoplasmic capsid assembly/packaging*				VZ-1950	GO:0039709		0
*> Nuclear capsid assembly*				VZ-1516	GO:0039708		10
Viral budding	KW-1198	212	15095	VZ-1947	GO:0046755	15'307	31'507
*> Viral budding via the host ESCRT complexes*	KW-1187	212	15095	VZ-1536	GO:0039702	15'307	15'928
*> Viral budding via viroporin*				VZ-5898			
*> Viral budding from Golgi membrane*				VZ-5900	GO:0046760		14
*> Viral Budding from ER membrane*				VZ-5899	GO:0046762		6
*> Viral budding from plasma membrane*				VZ-5901	GO:0046761		26
*> Viral Budding from nuclear membrane*					GO:0046765		8
Actin-dependent outward viral transport				VZ-5896			
Microtubular outwards viral transport	KW-1189	1	0	VZ-1816	GO:0039701	1	4
*> Cytoplasmic viral factory*					GO:0039714		40
*> Nuclear viral factory*					GO:0039715		0
Nuclear exit				VZ-2177	GO:0039674		21
*> Nuclear pore export*				VZ-1953	GO:0039675		0
*> Nuclear egress*	KW-1181	13	0	VZ-1952	GO:0046802	13	21
*> Nuclear envelope breakdown*				VZ-2176	GO:0039677		0
Viral occlusion body	KW-0842	44	466	VZ-1949	GO:0039679	510	510
Viral movement protein	KW-0916	306	620	VZ-1018	GO:0046740	926	2'941
Capsid maturation	KW-0917	122	135	VZ-1946	GO:0019075	257	392
Host cell lysis by virus	KW-0578	74	0	VZ-1077	GO:0044659	74	28
TOTAL		12'845	2'335'703			2'348'548	5'864'073

The table lists the 82 terms of the viral vocabulary as cited in the text. New terms created during this work in the three databases have been indicated by a grey background. The accession numbers are indicated for UniProtKB Keywords KW-XXX, ViralZone pages VZ-XXX and GO terms GO:XXXXXXX. The other columns indicate the number of annotations performed with this vocabulary/ontology. The SwissProt and TrEMBL columns display the number of annotations made using the corresponding KW in respectively reviewed and not reviewed UniProtKB entries. UniProt2GO column lists the number of annotation automatically mapped from UniProt to GO using the KW and GO term correspondence. GO annotation lists the total number of annotation using the corresponding GO term. KW in parentheses indicates terms for which the ontology used in UniProt applies to the product of a process, whereas in GO it refers to the molecules catalyzing it.

### Mapping of viral life-cycle processes to GO

The GO team at the EBI collaborated with the UniProtKB/SwissProt team to update and complete the GO database with the virus life-cycle molecular processes. The mapping effort led to the update of 56 GO terms and the development of 14 new GO terms (Table 1). 58 of those are directly related to ViralZone vocabulary, and reciprocally linked in ViralZone and GO pages [2]. The ViralZone vocabulary does not exactly match GO ontology, because the first provides knowledge in a web resource, while the second defines concepts/classes used to describe gene function, and relationships between these concepts. For example the page “Viral factories” (VZ-1951) in ViralZone describes all known features of this kind in one page. In GO this led to the creation of three terms: “viral factory” (GO:0039713), “cytoplasmic viral factory” (GO:0039714), and “nuclear viral factory” (GO:0039715). Other terms like “Nested subgenomic transcription” (VZ-1876) is a process that cannot yet be associated with a gene function and therefore did not lead to the creation of an associated GO term.

### Creation of new UniProtKB/Swiss-Prot keywords

**Fig 1:**
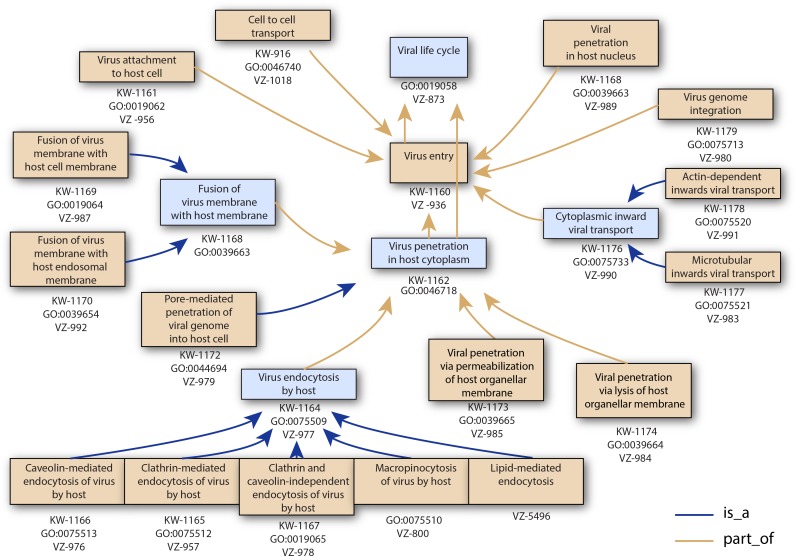
Example of ontology parent-child relationship. This tree consists of terms used to annotate the entry step of viral genomes. ViralZone pages (VZ), UniProtKB keyword (KW) or GO term accession numbers (GO:) are indicated. The hierarchy indicated is shared by GO and KW.

UniProtKB keywords summarize the content of a UniProtKB entry and facilitate the search for proteins of interest. Using ViralZone vocabulary we created 30 keywords (KW) and updated 11 KW (Table 1) for a total of 40. The keywords were developed in the case where several different viruses do use a common process, and can be linked to an individual protein’s functions. Therefore terms like “microtubular transport” were coined to annotate viral protein whose function is to trigger the transport, not to all the viral proteins actually transported by microtubules. 32 keywords on this list are linked to GO terms in UniProtKB, ViralZone and GO databases. These links allow automatic GO annotation based on UniProtKB KW through UniProtKB-Keyword2GO associations. UniProtKB KW can also describe the way proteins are produced, for example the “RNA editing” KW does not refer to proteins whose function is related to this process, but to proteins produced through this process. In Table 1 the accession numbers of these types of KW have been put in parentheses. They are not linked to GO terms, because “Viral RNA editing” (GO:0075527) is related to genes involved in the process of editing RNA, not produced by RNA editing. UniProtKB KW and GO terms are organized in a hierarchy, an example of which is pictured in Fig 1 for virus entry.

### Viral gene product curation with the new ontology

To demonstrate the utility of a standard ontology for virus biology, this work was completed by annotating virus data in the ViralZone, UniProtKB and Gene Ontology databases. Expert curation has been done in different ways. In UniProtKB/Swiss-Prot, keywords were manually introduced in viral entries after careful reading of the literature, using an editor available only to UniProtKB curators. All keywords with a related GO term (Table 1) were automatically annotated in GO through UniProtKB-Keyword2GO procedure. Moreover, expert curators manually associated GO terms to entries and publications with the Protein2GO editor. The latter is a web-based editor which can be used by any GO curators. Note that both UniProtKB and GO manual curations have a quality check to ensure the relevance of the information added. As of May 2016 the 40 UniProtKB/Swiss-Prot Keywords have now been manually associated 12,845 times to proteins, and automatically associated 2,335,703 times. The GO terms for viral life-cycle have been associated to genes 5,864,073 times. This number is high because many annotations already existed in GO for the 56 pre-existing viral life-cycle terms.

## Results

This works follows the events describing the fate of viral genetic material during the three stages of the infectious cycle: entry, genome expression/replication, and exit.

Virus entry starts with virion attachment to the host cell, leading to the uptake of the viral nucleic acid into a target cellular compartment in which it will start transcribing and replicating. The second step is transcription of viral genes, leading eventually to replication of the viral genome. Latency consists in a pause at the start of the transcription step; the viral genome is either silenced or transcribes few genes, putting on hold the resolution of the transcription/replication step. When this hold is released, the viral genome proceeds to the completion of this second step without going back to latency. The last step is virus assembly and exit. This corresponds to late transcription in most viral genomes. Often the virus will overproduce genomic and structural materials to assemble as many virions as possible. This can lead to irreversible damage to the host cell.

In the following paragraph, viral processes discussed in the text are underlined when they correspond to a vocabulary or ontology term. The corresponding ViralZone pages can be retrieved by typing the start of the term in the ViralZone search box and choosing the right name.

### Virus entry

**Fig 2:**
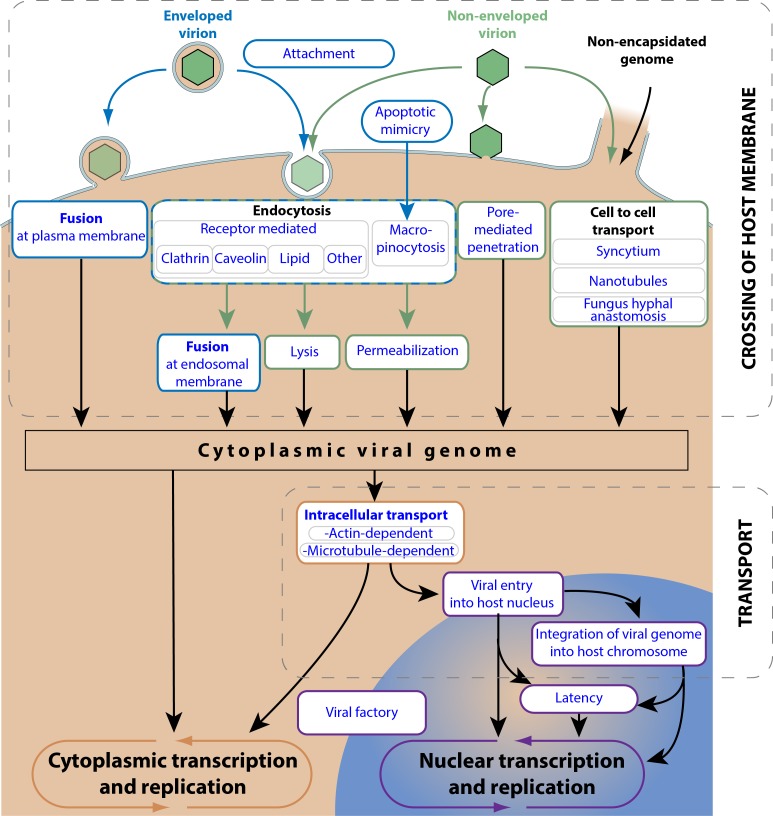
Entry pathways of eukaryotic viruses. This picture represents all the ViralZone controlled vocabularies concerning the virus entry pathway. The representation of viral entry is chronological. The virus genome begins on the top of the picture and will follow alternative pathways until reaching transcription/replication processes.

“Virus entry” refers to all the steps happening between the extracellular virion up to the transport of viral genetic material to the site of transcription/replication (Fig 2) [8]. The virus genome begins on the top of the picture and will follow alternative pathways until reaching the transcription/replication processes. The nature of the virus particle plays a decisive role in the routes of entry: enveloped viruses do not face the same challenges as non-enveloped capsids or even capsid-less viruses.

Viruses can infect new cells by many means. Some viruses exploit “cell to cell transport”. This includes plant plasmodesmata [9], nanotubules [10], fungus hyphal anastomosis [11] and syncytium formation [12]. The advantage of this kind of propagation is that the virus does not have to protect its genetic material by a capsid, or to exit from the infected cell. However it does not allow to jump from an animal or plant host to another, and target cells can only be those almost touching the previously infected cell.

The most classical route of infection is through an external virion particle that has to cross the cellular membrane to deliver its genetic material into the cell. The very first step is “viral attachment to host cell”, by binding surface molecules such as glycans or proteins [13]. Attachment is characterized as being reversible, as the interaction does not directly trigger internalization of the virus. The attachment step brings the virion closer to the host membrane where it can interact with an entry receptor. This receptor can be a host protein, a glycan or even lipids. Interaction with the entry receptor is not reversible because it triggers either “viral penetration in host cytoplasm” by “fusion of virus membrane with host cell membrane” (enveloped viruses) [14], “pore mediated penetration” (non-enveloped viruses) [15], or the uptake of virion particle “virus endocytosis by host” [16].

Endocytosis is an event whereby virion interaction with an entry receptor triggers active uptake of the virion by the cell to be brought to endosomes. The virus exploits an existing endocytic pathway to gain access to cellular internal compartments in early endosomes, late endosomes or even lysosomes from where it will be able to inject its genetic material into the cytoplasm. The nature of the host entry receptor bound by a virion likely determines which of the many routes of endocytosis it will use. There are four major routes: “clathrin-mediated endocytosis”, “caveolin-mediated endocytosis”, “lipid-mediated endocytosis” and “macropinocytosis” [16]. Interestingly the latter route can be triggered by “apoptotic mimicry”, a process in which an enveloped virus displays phosphatidyl serine at the surface of its membrane, thereby mimicking apoptotic bodies that are specifically macropinocytosed by dendritic or macrophage cells [17].

The endocytosed virion will then deliver its genetic material into the host cytoplasm often by exploiting the low pH endosomal environment. Enveloped virions will trigger “fusion of virus membrane with host endosomal membrane” [18], non-enveloped virions will induce “viral penetration via lysis of endosomal membrane” or “viral penetration via permeabilization of endosomal membrane”.

The viral genetic material delivered into the host cell cytoplasm is often addressed to a specific cellular location, either by “actin-dependent inwards transport” or “microtubule dependent inward transport” [19]. This transport is triggered by viral proteins bound to the viral genome. Nuclear viruses have a second barrier to cross: the nuclear membrane. They use either the nuclear pore at which the viral genetic material can be actively injected from the viral capsid (herpesviruses), or exploit nuclear import machinery (influenzavirus) [20]. A noteworthy variation of “viral penetration in host nucleus” is by infecting a cell during mitosis, when chromosomes are actually accessible from the cytoplasm without being protected by a nuclear membrane. This is the way many animal retroviruses infect cells, and thereby they can only infect dividing cells. Retroviruses finish their entry step by “viral genome integration” into the host chromosome. This can also happen occasionally for some parvovirus and herpesviruses.

At the end of virus entry step, the virus genome can either start transcribing/replicating leading to the formation of new progeny, or it may enter a latency mode. This mode is characterized by very low transcription of latent genes. The virus can stay dormant in the host cell for years before being activated by an external event [21].

### Virus genome expression and replication

**Fig 3:**
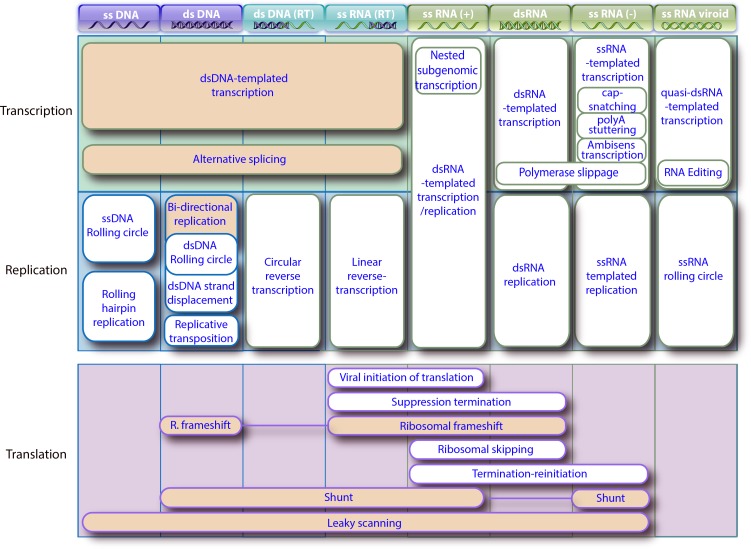
Viral specific transcription, replication and translation processes. This table lists all specific viral processes involved in transcription, replication or translation processes. The processes with orange backgrounds are also naturally used by eukaryotic cells, the others are specifically viral. All the processes are classified by the Baltimore classification (top row) which describes the nature of viral genome in the virion.

Viral genome expression is the second step of the infectious cycle, which often precedes “viral replication”. The nature of the genome is the critical point that determines the mechanism of transcription and replication. Therefore we have represented the different genetic expression/replication processes using the Baltimore classification (Fig 3) [22]. This classification separates viruses in seven groups depending on their genome architecture and their method of replication: single stranded DNA (ssDNA), dsDNA, dsDNA reverse transcribing (dsDNA RT), ssRNA reverse transcribing (ssRNA RT), positive-stranded ssRNA (ssRNA+) and negative stranded ssRNA (ssRNA-). We have added an eighth class for ss/dsRNA viroids and hepatitis delta which have very specific means of transcription/replication. Some viruses during replication/transcription assemble a dedicated cellular compartment called “viral factories” [23].

Viral dsDNA templated transcription is performed by classical cellular mechanisms, or the viral equivalent of it. To improve coding capacity, cellular splicing is exploited by dsDNA viruses that transcribe in the host nucleus. There are at least seven ways to replicate the genome of viruses having a dsDNA intermediate. The classical cellular “bi-directional replication” (papillomavirus, polyomavirus) [24] can be replaced by viral “dsDNA rolling circle” (herpesvirus) [25], “ssDNA rolling-circle” (circovirus) [26], “dsDNA strand displacement” (adenovirus) [27], or retro-transcription in the case of dsDNA(RT) and ssRNA(RT) viruses [28]. Many ssDNA or dsDNA viruses replicate in the nucleus by highjacking the cellular machinery (papillomaviruses) [24], or using a mix of cellular and viral enzymes (herpesviruses) [25]. But cytoplasmic DNA viruses (poxviruses, mimiviruses) encode entirely for their own transcription and replication machinery [29].

Ss(+)RNA and dsRNA viral genomes are transcribed by viral RNA-dependent RNA polymerases from a dsRNA template. Interestingly, “ss(+)RNA replication” and transcription are similar, in that the same genomic mRNA is the template for translation and replication.

Within eukaryotic cells, dsRNA is a strong inducer of antiviral-defense. Therefore RNA viruses hide their dsRNA template or prevent its formation: ss(+)RNA virus transcription/replication happens in membranous vesicles [30], whereas “dsRNA replication” is hidden in icosahedral capsid [31]. “ss(-)RNA replication” is noteworthy because both viral genomes and antigenomes are tightly covered with nucleocapsids to prevent their annealing and the formation of dsRNA [32]. ss(-)RNA genome transcriptase uses a single stranded RNA as template; this is the only known transcription performed from single stranded nucleic acid, and requires that nucleoprotein cover the single-stranded RNA template [33]. This unique transcription is associated with unique mechanisms to produce bona fide mRNA: the “Cap snatching” consists of using a cut off host mRNA CAP to initiate transcription [34], and “Poly A stuttering” to produce a non-templated polyA tail [35]. Paramyxoviruses and filoviruses can also enhance their coding capacity by a unique co-transcriptional “RNA editing” process, also called polymerase slippage [36].

Viroids and the hepatitis delta RNA genome consist of a partially double-stranded closed circular RNA molecule. Interestingly, “Viroids and hepatitis D replication” and “hepatitis D transcription” are assured by the host DNA dependent RNA polymerase, that is exceptionally able under these circumstances to use a RNA template [37].

After replication/ transcription, viral mRNA is translated to produce viral proteins, but no known virus encodes for any translation machinery. Indeed, viruses can be defined as replicative genetic elements that do not encode ribosomes. The absence of a translation system is what defines their very parasitic nature. Therefore, viral translation is performed by host cellular machinery, and follows classical cellular mechanisms. Nonetheless, viruses trick host ribosomes in many ways to enhance the protein expression from their small genomes. This includes: “leaky scanning” [38], “ribosomal frameshift” [39], “suppression of termination” [40], “ribosomal skipping” [41], “termination-reinitiation” [42]; and “viral initiation of translation” whereby viruses bypass the need for a mRNA CAP for efficient translation [43].

### Virus exit from host cell

**Fig 4:**
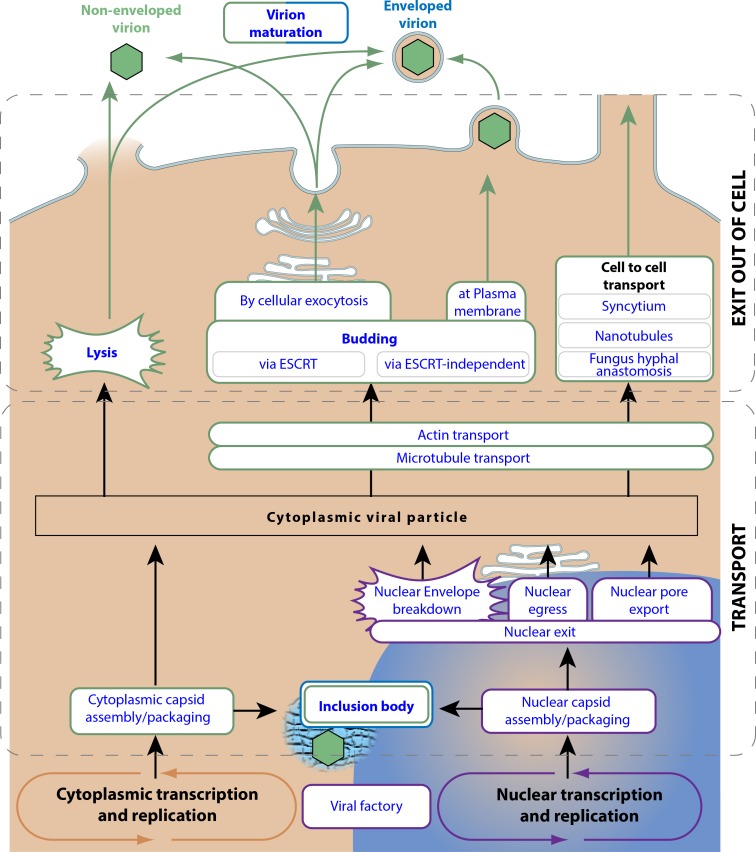
Exit pathways of eukaryotic viruses. This picture represents all the ViralZone controlled vocabularies concerning the virus exit pathway. The representation is chronological: The virus genome begins at the bottom of the picture at transcription/replication processes and will follow alternative pathways until exiting the host cell at the top of picture.

After the replication phase, viruses express movement and/or structural proteins as means to export their genomes out of the cell (Fig 4). “Viral movement proteins” allow viruses to exploit cell to cell transport, thereby infecting new cells without actually exiting out of host cytoplasm. This can happen through syncytium (poxvirus) [12], nanotubules (HIV) [10], plant plasmodesmata [9] or fungus anastomosis [11]. But these bridges are seldom available between hosts, and viruses must find a way to exit the cell’s environment to be able to infect other cells. Therefore most viruses produce virions that will protect their fragile genome outside of the infected cell. For this, the viral genome needs to be properly packaged and encapsidated with structural proteins.

The easiest way for a virus to exit the host cells it to induce its death or lysis. This can occur naturally as for corneocytes (papillomaviruses) [44], or be induced by “host cell lysis by virus” (polyomaviruses) [45]. In some cases, the host cell dies by being filled with “occlusion bodies” that will later protect virions in the environment (poxviruses, baculoviruses) [46]. Although highly efficient, lytic destructive behavior can be a handicap in multicellular organisms and trigger unwanted immune system activation. Therefore, many eukaryotic viruses have evolved to bud from an infected cell without lysing it.

To physically exit from the cell, the viral particle or genome have to be transported to the plasma membrane or to the cellular exocytosis machinery. Nuclear virus genomes migrate to the cytoplasm by “nuclear pore export” (influenza, HIV) [47], or budding out of the nuclear membrane through a mechanism called “nuclear egress” (herpesviruses) [48]. Cytoplasmic viral particles can be targeted by actin or microtubule outward transport to the appropriate place for budding/exit [49,50]. “Viral budding” takes place at the endoplasmic reticulum (picornavirus) [51] or the Golgi (herpesviruses) [52] to expel the viral particle by exocytosis, or happens directly at the plasma membrane (filovirus) [53]. Enveloped viruses acquire a cell-derived envelope upon budding. They exploit either the endosomal sorting complexes required for transport (ESCRT) machinery (rabies virus) [54], or a process involving viroporins which is called ESCRT-independent budding (influenzavirus) [55]. After viral particle release out of the cell, a last step can involve “capsid maturation”, as occurs for retroviruses in which the GAG-POL polyprotein are cleaved into several chains [56]. The mature viral particle is called a virion, and is ready to infect a new host.

### Viral ontology applications

The first application of the viral ontology is to allow comprehensive annotation of virus genes and sequences in databases. Moreover, developing an ontology is akin to defining a set of data and their structure for other programs to use. Computers programs can use ontologies as data in any of their analysis. Therefore, the viral ontology gives computers access to a kind of expert knowledge analysis that can be essential in research. For example, Brandes et al. have recently used ViralZone capsid ontology data in their statistical analysis about gene overlapping and size constraints in the viral world [57]. Moreover with the advent of large scale technologies comprehensive ontologies are essential to associate knowledge with large-scale data by computer analysis [58].

## Discussion

The virus replication cycle vocabulary and ontology have been expanded by collaboration between the Swiss-Prot and GO teams. These vocabulary and ontologies are all linked together and describe the mechanisms involved in eukaryotic viruses’ life-cycles. While most of our current knowledge is covered by these terms, our systematic approach will allow for expanding and updating the system. One achievement of this work is that it allows a virus’ life-cycle to be described by a succession of controlled vocabularies. This provides a means to store and manage knowledge in biological databases. For example, Zika virus life-cycle can be summarized by cutting this cycle into steps described by controlled vocabulary: “Attachment”, “Apoptotic mimicry”, “Viral endocytosis/ macropinocytosis”, “Fusion with host endosomal membrane”, “Viral factory”, “dsRNA-templated transcription/replication”, “Cytoplasmic capsid assembly”, “Viral budding via the host ESCRT complexes”, “Virus budding by cellular exocytosis”. These successions of terms describe accurately the pathway followed by the Zika virus genome across an infected cell. It uses ViralZone controlled vocabulary because some processes cannot be described by GO or UniProtKB ontologies when they cannot be associated with a gene. For example “Apoptotic mimicry” cannot be related to a viral gene or protein, as it involves the virion membrane.

Our efforts to create a eukaryotic virus ontology have led to three levels of implementation: global knowledge and facts in ViralZone pages; viral protein annotation in UniProtKB through keywords; and viral gene and protein annotation through GO terms. This has led to the creation of 69 new ViralZone pages, at least 30 new SwissProt keywords and 59 new GO terms. At the time of writing (May 2016) the keywords provide a total of 2,348,548 annotations in UniProtKB while the equivalent GO terms provide 5,864,073 annotations. Together these three implementations provide a global view of viral biology, and a means to annotate knowledge, for a wide user community. Research groups may contribute to this viral ontology by providing suggestions for updating terms (e.g. requests for new terms) either through ViralZone (viralzone@isb-sib.ch) or Gene Ontology (http://geneontology.org/contributing-go-term). Several research institutes and public databases have initiated projects involving the annotation of viral genomes, and we hope that the terms and ontologies presented in this article, which are available from the ViralZone, UniProtKB and GO websites, will help them in these efforts.
